# Hybrid MICO-LAC Segmentation with Panoptic Tumor Instance Analysis for Dense Breast Mammograms

**DOI:** 10.3390/jimaging12030095

**Published:** 2026-02-24

**Authors:** Razia Jamil, Min Dong, Orken Mamyrbayev, Ainur Akhmediyarova

**Affiliations:** 1 School of Electrical and Information Engineering, Zhengzhou University, Zhengzhou 450001, China; 2 Institute of Information and Computational Technologies, Almaty 050010, Kazakhstan; 3 Institute of Automation and Information Technologies, Satbayev University, Almaty 050013, Kazakhstan

**Keywords:** breast cancer segmentation, mammography, MICO, mumford shah model, localized active contours, dense breast tissue

## Abstract

This study proposes a clinically driven hybrid segmentation framework for dense breast tissue analysis in mammographic images, addressing persistent challenges associated with intensity inhomogeneity, low-contrast, and complex tumor morphology. The framework integrates Multiplicative Intrinsic Component Optimization (MICO_2D) for bias field correction, followed by a distance-regularized multiphase Vese–Chan level-set model for coarse global tumor segmentation. To achieve precise boundary delineation, a localized refinement stage is employed using Localized Active Contours (LAC) with Local Image Fitting (LIF) energy, supported by Gaussian regularization to ensure smooth and coherent boundaries in regions with ambiguous tissue transitions. Building upon the refined semantic tumor mask, the framework further incorporates a panoptic-style tumor instance segmentation stage, enabling the decomposition of connected tumor regions into distinct anatomical instances, which were evaluated on both MIAS and INBreast mammography datasets to demonstrate generalizability. This extension facilitates detailed structural analysis of tumor multiplicity and spatial organization, enhancing interpretability beyond conventional pixel wise segmentation. Experiments conducted on Cranio-Caudal (CC) and Medio-Lateral Oblique (MLO) mammographic views demonstrate competitive performance relative to baseline U-Net and advanced deep learning fusion architectures, including multi-scale and multi-view networks, while offering improved interpretability and robustness. Quantitative evaluation using overlap-related metrics shows strong spatial agreement between predicted and reference segmentations, with per-image Dice Similarity Coefficient (DSC) and Intersection over Union (IoU) distributions reported to ensure reproducibility. Descriptive per-image analysis, supported by bootstrap-based confidence intervals and paired comparisons, indicates consistent performance improvements across images. Robustness analysis under realistic perturbations, including noise, contrast degradation, blur, and rotation, demonstrates stable performance across varying imaging conditions. Furthermore, feature space visualizations using t-SNE and UMAP reveal clear separability between cancerous and non-cancerous tissue regions, highlighting the discriminative capability of the proposed framework. Overall, the results demonstrate the effectiveness, robustness, and clinical motivation of this hybrid panoptic framework for comprehensive dense breast tumor analysis in mammography, while emphasizing reproducibility and conservative statistical assessment.

## 1. Introduction

Breast cancer remains one of the most prevalent malignancies worldwide. According to 2022 GLOBOCAN estimates, approximately 2.3 million women were diagnosed with the disease, resulting in about 670,000 deaths globally [1]. In the United States alone, projections for the coming years anticipate around 310,000–320,000 new cases of invasive breast cancer in women, roughly 2700 cases in men, and approximately 56,000–60,000 cases of ductal carcinoma in situ (DCIS) in women [2]. As a leading cause of cancer-related mortality among women, breast cancer poses a significant public health challenge [3]. Although the disease affects individuals across demographics, risk varies substantially by age and gender, with incidence rising sharply in older age groups. The age-standardized incidence rate in women has shown a consistent upward trend, with a projected annual percentage change of approximately 0.44 from 2020 to 2030 [4]. Mammography continues to serve as the primary modality for early detection. However, interpretation is frequently complicated by dense breast tissue, low contrast, and intricate structural patterns [5]. These factors heighten dependence on expert radiologists and contribute to inter-observer variability, potentially leading to missed or inconsistent diagnoses. Accurate lesion segmentation is therefore critical for computer-aided detection (CAD) systems and informed clinical decision-making [6]. Yet segmentation remains difficult owing to inter-patient variability in anatomy, tissue heterogeneity, and differences in image acquisition positioning. To address these limitations, this study introduces a hybrid segmentation framework designed as a decision-support tool for analyzing dense mammograms. The approach integrates established optimization-based techniques in a sequential, structured pipeline: clustering-based intensity correction, distance-regularized multiphase Vese–Chan global segmentation, and a two-stage localized refinement using active contours with local image fitting (LIF) and Gaussian regularization. This combination aims to achieve precise tumor boundary delineation, even in challenging low-contrast and heterogeneous tissue environments. A key focus of this work is to determine whether a training-free, purely mathematically driven framework can provide reliable tumor segmentation under conditions of low contrast and tissue complexity, and whether a deterministic panoptic-style instance separation strategy can yield meaningful structural insights without depending on data-driven deep learning models. The proposed framework is rigorously compared against representative multi-scale and multi-view deep learning baselines, particularly under limited-data scenarios, using both craniocaudal (CC) and mediolateral oblique (MLO) views. The main contributions of this work are summarized as follows:

- A clinically driven hybrid segmentation framework for accurate delineation of dense breast tumors in mammograms.
- Integration of MICO_2D for intensity inhomogeneity correction and bias aware initial segmentation.
- A Distance-Regularized (DR) multiphase Vese–Chan model for robust global tumor region extraction.
- A two-stage localized refinement strategy combining LAC and LIF for precise boundary delineation.
- A panoptic-style instance segmentation extension enabling separation of connected tumor regions for improved structural interpretation.
- Comparative evaluation against multi-scale and multi-view deep learning baselines using CC and MLO views.
- Comprehensive robustness analysis using image perturbations, uncertainty estimation, and feature space visualization (t-SNE and UMAP).

The remainder of this paper is organized as follows. Section 2 reviews related literature on mammographic segmentation and its limitations. Section 3 presents the proposed methodology in detail, including preprocessing, global segmentation, and refinement stages and panoptic segmentation. Section 4 describes the experimental setup, datasets, evaluation metrics, and quantitative and qualitative results. Finally, Section 5 concludes the paper and outlines future research directions.

## 2. Related Work

Mammographic image segmentation plays a pivotal role in automated breast cancer detection by accurately identifying regions of interest (ROIs), such as tumors or microcalcifications. However, the task is complicated by inherent challenges, including image noise, overlapping anatomical structures, low contrast, and intensity inhomogeneities caused by varying breast density and acquisition artifacts. These issues often degrade segmentation performance and increase false positives or negatives, prompting extensive research into preprocessing, segmentation, and refinement strategies.

### 2.1. Preprocessing Techniques

Effective preprocessing is essential to enhance mammogram quality, correct intensity variations, and improve tumor visibility for subsequent analysis. Traditional approaches include histogram-based methods such as Contrast-Limited Adaptive Histogram Equalization (CLAHE) and Adaptive Histogram Equalization (AHE), which enhance local contrast while mitigating over-amplification of noise [7]. Clustering algorithms like K-means and Fuzzy C-Means (FCM) have been widely adopted for bias field correction and intensity inhomogeneity mitigation. More advanced techniques leverage fractional calculus, such as Grünwald–Letnikov derivative masks, to adaptively deblur images, yielding superior Peak Signal-to-Noise Ratio (PSNR), Absolute Mean Brightness Error (AMBE), and entropy compared to conventional filters [8]. Other notable methods include Non-Local Means (NLM) filtering for noise reduction, Gray-Level Run-Length Matrix (GLRLM) for texture enhancement, Brightness Preserving Gradient-Related Joint Histogram Equalization (BPGJHE) to preserve structural details and brightness [9], and optimized frameworks like OKF-AGCWD, which integrates differential evolution, kernel FCM, and cuckoo search optimization for enhanced contrast and downstream segmentation accuracy [10]. These preprocessing steps consistently improve the reliability of feature extraction and detection, particularly in dense breast tissue. Early deep-learning-based preprocessing and classification efforts, such as CNNs on Mini-MIAS datasets achieving  62% accuracy, have evolved toward hybrid models. For instance, K-NN combined with Kernel-Based CNN (KBCNN) has reported over 95% accuracy in tumor classification [11]. Models like Bi-CBMSegNet, which fuse global and local feature extraction, have demonstrated Dice scores exceeding 97% on DDSM and INbreast datasets, outperforming many standalone segmentation approaches [12].

### 2.2. Segmentation Methods

Segmentation techniques for mammograms are broadly categorized as threshold-based, region-based, or model-based. Thresholding methods, such as Otsu’s algorithm, offer computational efficiency but remain sensitive to noise and intensity variations. Region-growing and watershed algorithms incorporate spatial context yet are prone to over-segmentation in heterogeneous tissues.

Level-set methods have emerged as robust alternatives, with the classic Mumford–Shah functional [13] serving as a foundation for many variants. Enhancements include Transformed Total Variation (TTV) regularization with $\ell_{1}$ norms for improved edge recovery and noise resilience [14], fuzzy membership integration via Alternating Direction Method of Multipliers (ADMM), and multiphase extensions optimized by metaheuristics like Cuckoo Search to handle both homogeneous and inhomogeneous regions [15]. Other innovations include the SaT (Smoothing and Thresholding) technique and Bayesian inference for parameter optimization, reducing computational demands while maintaining accuracy [16].

Deep learning architectures have advanced segmentation significantly. MAU-Net integrates mixed depth-wise convolutions, context pyramid modules, and self-ensemble decoding to capture multi-scale spatial features, delivering state-of-the-art Dice scores on datasets like BraTS [17]. Cascaded models incorporating saliency maps and attention mechanisms reduce false negatives and improve performance on INbreast, CSAW-S, and DMID datasets [18]. Hybrid CAD pipelines combining XGBoost classification, EfficientNetB0, hybrid CNNs, and Attention U-Net have achieved 96.14% abnormal and 94.81% cancer classification accuracy on BUSI [19], while architectures like ACA-ATRUNet and MML-EOO further refine early screening and boundary precision [20].

Refinement stages address imprecise or weak boundaries by leveraging edge and region information. Classic active contour models (Snakes) [21] and geodesic active contours [22] effectively handle complex topologies. Local Image Fitting (LIF) energy with Gaussian regularization excels in inhomogeneous regions by incorporating local intensity statistics [23], while hybrid LIF combined with Difference of Gaussians (DoG) reduces iteration counts and sharpens edge detection.

Hybrid approaches that combine preprocessing, segmentation, and refinement yield superior overall performance. CNN-based hybrids on Wisconsin Breast Cancer and CBIS-DDSM datasets achieve high AUC-ROC, sensitivity, specificity, and accuracy [24]. Deep learning–machine learning integrations mitigate computational overhead while boosting accuracy [25], and feature fusion strategies incorporating Gabor, Prewitt, GLCM, and CNNs improve microcalcification detection to 89.56% [26,27]. These frameworks underscore the value of multi-stage pipelines in overcoming mammographic challenges. Table 1 provides a comparative summary of the key methods’ performance across common mammogram datasets, highlighting trade-offs in accuracy, robustness, and computational efficiency.

Recent hybrid and attention-related methodologies, exemplified as a hybrid attention network for precise breast tumor segmentation in ultrasound images [41], have exhibited enhanced boundary delineation and feature representation, underscoring the promise of therapeutically driven hybrid tactics. In contrast to existing methodologies, our framework is devoid of training, interpretable, and specifically designed for the segmentation of dense mammographic tissue, integrating preprocessing, multiphase level-set segmentation, and localized refinement. USE-MiT utilizes a UNet backbone, linking the encoder and decoder with Squeeze-and-Excitation Attention modules, with the encoder incorporating a Mix Transformer, resulting in a Dice score of 0.88 and an IoU of 0.64 on benchmark datasets [42]. SwinEff-AttentionNet incorporates Swin transformers, EfficientNet layers, and Efficient Local Self-Attention (ELSA) modules, attaining Dice scores of 0.92 and 0.878, as well as IoU scores of 0.887 and 0.83 on the BUSI and Breast-Lesions-USG datasets. These strategies underscore the efficacy of hybrid attention processes in enhancing segmentation performance and addressing complex ultrasound images [43].

To address these challenges, this paper proposes a novel hybrid framework for breast cancer detection in mammograms, combining advanced preprocessing, segmentation, and refinement techniques. Key contributions include: MICO_2D for initial segmentation, an improved Vese–Chan model with DR for robust segmentation, and a two-stage refinement process using LAC and LIF for sub-pixel precision. Experimental results show the method outperforms existing approaches in terms of DSC and IoU, with statistical significance confirmed by t-statistics and bootstrap confidence intervals.

## 3. Materials and Methods

**Figure 1:**
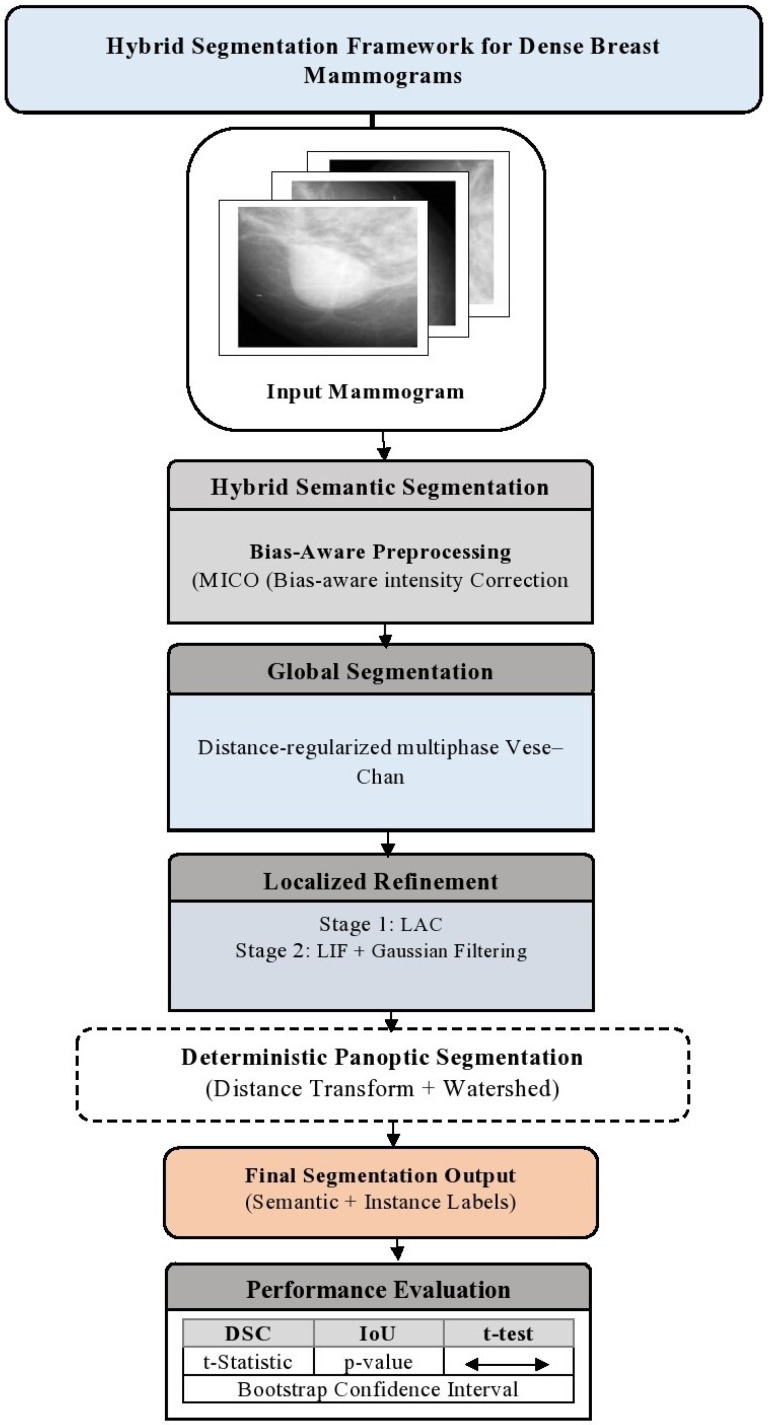
Overview of the proposed hybrid MICO–LAC panoptic segmentation framework, showing the sequential, dependent processing stages.

The proposed framework for breast cancer detection in mammographic images consists of three primary stages: preprocessing, global segmentation, and localized refinement. Figure 1 illustrates the overall framework of the proposed system. Although each component of the framework is well established, their integration is non-trivial in the context of dense mammography. Challenges such as intensity inhomogeneity, weak tumor boundaries, and heterogeneous tissue distributions necessitate a carefully structured workflow to prevent error propagation. Accordingly, the proposed methodology addresses these challenges through bias-aware preprocessing, globally stable contour evolution, and targeted localized refinement.

### 3.1. Image Preprocessing

Prior to segmentation, mammograms underwent a preprocessing pipeline that included breast region extraction and background removal to eliminate non-breast structures (e.g., labels, artifacts). Images were then normalized to a standard intensity range, and MICO-based bias correction was applied to reduce intensity inhomogeneity. These steps ensured consistent input across cases, reduced noise-related artifacts, and stabilized subsequent contour evolution.

### 3.2. Dataset and Experimental Setup

The proposed methodology is evaluated using the publicly available MIAS dataset [44], comprising 322 mammograms with malignant, benign, and normal dense breast cases. Images were digitized at 50-µm resolution and resampled to 200-µm with standardized 1024×1024 dimensions for both CC and MLO views. To assess generalizability, the framework was also evaluated on the INBreast dataset [45], which includes 410 high-resolution mammograms with malignant, benign, and normal cases. Segmentation performance was measured using the Dice Similarity Coefficient (DSC), boundary accuracy, and computational efficiency. MIAS and INBreast were chosen for their standardized acquisition protocols and reliable expert annotations, enabling meaningful evaluation and comparison with prior studies. The framework is mathematically driven and training-free, processing each image independently without train/validation/test splits. Level-set functions are initialized using signed distance maps from the bias-corrected MICO output, ensuring stable contour evolution. Experiments were implemented in MATLAB R2021a (64-bit) on an Intel Core i7 CPU with 8 GB RAM, and ground truth masks were verified with interactive annotation tools. Although MIAS is smaller and older, it remains a standard benchmark. All comparative deep learning baselines were evaluated under identical data conditions, without external datasets or augmentation, ensuring fair comparison. As the framework is resolution-agnostic and parameter-free, it is expected to generalize to modern digital mammograms. Future work will extend validation to larger, multi-institutional datasets.

### 3.3. Proposed Architecture Overview

This hybrid framework addresses common challenges, including intensity inhomogeneities, ambiguous boundaries, and noise in mammograms, by integrating multiple advanced techniques to accomplish precise segmentation. The panoptic-style tumor instance segmentation stage operates on the refined semantic tumor mask and does not alter pixel-level tumor boundaries. Instead, it deterministically decomposes connected tumor regions into distinct anatomical instances. Consequently, this stage does not contribute to improvements in conventional segmentation metrics such as Dice or IoU, but rather enables instance-level structural analysis and enhanced clinical interpretability. Each component of the formulation serves a specific purpose; MICO_2D corrects intensity inhomogeneity, the multiphase level set provides robust global tumor separation and the localized refinement improves boundary precision in ambiguous regions. This hierarchical design balances robustness, interpretability and clinical relevance. The complete process is described in Algorithm 1.
**Algorithm 1:** Hybrid Panoptic-Style Segmentation of Dense Breast Tumors**Input**: Mammogram image I(x,y)**Output**: Panoptic segmentation map P(x,y)**Step 1: Preprocessing**Normalize the input image to obtain $I_{n}\left(x,y\right)$;**Step 2: Bias Correction**Perform intensity inhomogeneity correction using the MICO_2D model to obtain $I_{c}\left(x,y\right)=I_{n}\left(x,y\right)/b\left(x,y\right)$;**Step 3: Global Segmentation**Apply distance-regularized multiphase Chan–Vese segmentation to obtain a coarse semantic mask $S_{g}\left(x,y\right)$;**Step 4: Localized Refinement**Refine object boundaries using localized active contours with LIF energy to obtain $S_{r}\left(x,y\right)$;**Step 5: Instance Extraction**Extract tumor instances $\left\{T_{k}\right\}$ from $S_{r}\left(x,y\right)$ using distance transform and watershed segmentation;**Step 6: Panoptic Assignment**Assign semantic and instance labels to construct the panoptic output P(x,y);

### 3.4. Hybrid MICO-LAC Panoptic Segmentation Framework

To begin, the raw mammogram image, denoted as *I*, is considered as the input for preprocessing and subsequent analysis steps. The input image is defined as a discrete function of spatial coordinates as

(1) I(x,y),x,y∈Z.

The MICO_2D method is employed for preliminary segmentation of the input image. This process produces an initial segmented output $S_{0}$, which delineates the regions of interest and is expressed as

(2) $$S_{0}\left(x,y\right)={\mathrm{Segmentation}}_{\mathrm{MICO}\_2D}\left(I(x,y)\right).$$

The segmentation process minimizes the following energy functional:

(3) $$E\left(M,C,b\right)=\sum_{k=1}^{N_{\mathrm{class}}}\int_{\Omega }M_{k}^{q}{\left(I-C_{k}b\right)}^{2}\;d\Omega ,$$

where $M_{k}$ denotes the membership function of each region, $C_{k}$ represents the mean intensity of class *k*, *b* is the bias field, *q* is the fuzziness parameter, and Ω denotes the image domain. For q>1, the membership functions are updated according to

(4) $$M_{k}=\frac{{\left({\left(I-C_{k}b\right)}^{2}\right)}^{-\frac{1}{q-1}}}{\sum_{j=1}^{N_{\mathrm{class}}}{\left({\left(I-C_{j}b\right)}^{2}\right)}^{-\frac{1}{q-1}}}.$$

For the crisp segmentation case (q=1), the membership function is defined as

(5) $$M_{k}=\left\{\begin{array}{cc} 1, & \mathrm{if}\;k=\arg\min_{j}{\left(I-C_{j}b\right)}^{2},\\ 0, & \mathrm{otherwise}. \end{array}\right.$$

The class means are iteratively updated using

(6) $$C_{k}^{\mathrm{new}}=\frac{\sum_{\Omega }b\;I\;M_{k}\;W}{\sum_{\Omega }b^{2}M_{k}\;W}.$$

The bias field is modeled using a linear combination of basis functions:

(7) $$b=\sum_{k=1}^{N_{\mathrm{bas}}}w_{k}\;{\mathrm{Bas}}_{k},$$

where the coefficient vector is computed as

(8) $$w=A^{-1}V.$$

The energy function is evaluated at each iteration to ensure convergence:

(9) $$E\left(M,C,b\right)=\sum_{k=1}^{N_{\mathrm{class}}}M_{k}^{q}{\left(I-C_{k}b\right)}^{2}\;d\Omega .$$

#### 3.4.1. Intensity Inhomogeneity Correction

To correct intensity inhomogeneities, the input image is normalized to a fixed range:

(10) $$I^{\prime }\left(x,y\right)=A\cdot \frac{I(x,y)-\min(I)}{\max(I)-\min(I)}.$$

Two level-set functions are initialized as

(11) $$\mu_{1}\left(x,y\right)=\mathrm{sign}\left(M\left(x,y\right)-0.5\right),\;\mu_{2}\left(x,y\right)=\mathrm{sign}\left(M\left(x,y\right)-0.5\right).$$

These functions define three disjoint regions:

(12) $$M_{1}\left(x,y\right)=H\left(\mu_{1}\right)H\left(\mu_{2}\right),$$

(13) $$M_{2}\left(x,y\right)=H\left(\mu_{1}\right)\left(1-H(\mu_{2})\right),$$

(14) $$M_{3}\left(x,y\right)=1-H\left(\mu_{1}\right),$$

where H(·) denotes the Heaviside function. The bias field is initialized as

(15) b(x,y)=1.

The total energy functional is defined as

(16) $$\begin{array}{cc} E(\mu_{1},\mu_{2},b,C)=\; & \sum_{i=1}^{3}\int_{\Omega }{\left(b\left(x,y\right)I\left(x,y\right)-C_{i}\right)}^{2}M_{i}\left(x,y\right)\;dxdy\\ \; & +v\int_{\Omega }\left|\nabla H\left(\mu \right)\right|\;dxdy+\mu R\left(\mu_{1},\mu_{2}\right). \end{array}$$

The bias field is updated iteratively using

(17) $$b\left(x,y\right)=\frac{K*\left(I\left(x,y\right)\sum_{i=1}^{3}C_{i}M_{i}\left(x,y\right)\right)}{K*\sum_{i=1}^{3}M_{i}\left(x,y\right)},$$

where *K* denotes a Gaussian kernel. The evolution equations of the level-set functions are given by:

(18) $$\frac{\partial \mu_{1}}{\partial t}=\delta \left(\mu_{1}\right)\left[\nu \;\nabla \cdot \left(\frac{\nabla \mu_{1}}{|\nabla \mu_{1}|}\right)-\sum_{i=1}^{3}\left({\left(bI-C_{i}\right)}^{2}\;\frac{\partial M_{i}}{\partial \mu_{1}}\right)\right]$$

(19) $$\frac{\partial \mu_{2}}{\partial t}=\delta \left(\mu_{2}\right)\left[v\nabla \cdot \left(\frac{\nabla \mu_{2}}{|\nabla \mu_{2}|}\right)-\sum_{i=1}^{3}{\left(bI-C_{i}\right)}^{2}\frac{\partial M_{i}}{\partial \mu_{2}}\right].$$

After convergence, the segmented image is reconstructed as

(20) $$I_{\mathrm{segmented}}\left(x,y\right)=C_{1}M_{1}\left(x,y\right)+C_{2}M_{2}\left(x,y\right)+C_{3}M_{3}\left(x,y\right).$$

The bias-corrected image is computed by

(21) $$I_{bc}\left(x,y\right)=\frac{I(x,y)}{B(x,y)+\epsilon }.$$

#### 3.4.2. Global Segmentation Using Enhanced Vese–Chan with DR

Following bias correction, a refined Vese–Chan model with DR is employed for global segmentation. This model is derived from the Mumford–Shah functional and utilizes a level-set function ϕ(x) to partition the image into foreground and background regions. The corresponding energy functional is defined as

(22) $$E\left(\phi \right)=\lambda_{1}\int_{\Omega_{1}}{\left(I\left(x\right)-c_{1}\right)}^{2}dx+\lambda_{2}\int_{\Omega_{2}}{\left(I\left(x\right)-c_{2}\right)}^{2}dx+v\int_{\partial \Omega }\left|\nabla \phi \left(x\right)\right|dx,$$

where $\Omega_{1}$ and $\Omega_{2}$ denote the regions inside and outside the contour, respectively. The parameters $c_{1}$ and $c_{2}$ represent the average intensities within these regions, and *v* controls the boundary length penalty. The level-set evolution equation corresponding to this energy is given by

(23) $$\frac{\partial \phi (x,t)}{\partial t}=\delta \left(\phi \right)\left[\lambda_{1}{\left(I\left(x\right)-c_{1}\right)}^{2}-\lambda_{2}{\left(I\left(x\right)-c_{2}\right)}^{2}\right]+v\nabla \cdot \left(\frac{\nabla \phi (x)}{\left|\nabla \phi (x)\right|}\right).$$

To prevent numerical degradation of the level-set function during evolution, a DR term is incorporated, yielding the improved energy functional

(24) $$\begin{array}{cc} E(\phi )=\; & \lambda_{1}\int_{\Omega_{1}}{\left(I\left(x\right)-c_{1}\right)}^{2}dx+\lambda_{2}\int_{\Omega_{2}}{\left(I\left(x\right)-c_{2}\right)}^{2}dx\\ \; & +v\int_{\partial \Omega }|\nabla \phi \left(x\right)|dx+\mu \int_{\Omega }{\left|\nabla \phi \left(x\right)\right|}^{2}dx, \end{array}$$

where μ is the regularization coefficient enforcing the signed distance property of the level-set function. The corresponding evolution equation becomes

(25) $$\frac{\partial \phi (x,t)}{\partial t}=\delta \left(\phi \right)\left[\lambda_{1}{\left(I\left(x\right)-c_{1}\right)}^{2}-\lambda_{2}{\left(I\left(x\right)-c_{2}\right)}^{2}\right]+v\nabla \cdot \left(\frac{\nabla \phi (x)}{\left|\nabla \phi (x)\right|}\right)-\mu \nabla \cdot \left(\frac{\nabla \phi (x)}{\left|\nabla \phi (x)\right|}\right).$$

A global threshold *T* is then applied to the bias corrected image to generate the global segmentation mask:

(26) $$S_{g}\left(x,y\right)=\left\{\begin{array}{cc} 1, & \mathrm{if}\;I_{bc}\left(x,y\right)\geq T,\\ 0, & \mathrm{if}\;I_{bc}\left(x,y\right)<T. \end{array}\right.$$

#### 3.4.3. Localized Refinement Using Active Contours

To further improve boundary delineation, LAC models are applied to the global segmentation output. The Active Contour Mumford–Shah (ACMS) energy functional is expressed as

(27) $$E_{\mathrm{MS}}\left(C,u\right)=\int_{\Omega }{\left(f(x,y)-u(x,y)\right)}^{2}dxdy+\alpha \int_{\Omega }{\left|\nabla u\left(x,y\right)\right|}^{2}dxdy+\beta \;\mathrm{Length}\left(C\right),$$

where f(x,y) denotes the input image, u(x,y) is a piecewise smooth approximation, and *C* represents the evolving contour. In the localized formulation, the energy is computed within a neighborhood $\Omega_{r}$ around the contour:

(28) $$E_{\mathrm{Local}\_\mathrm{MS}}\left(C,u\right)=\int_{\Omega_{r}}{\left(K\left(x,y\right)\left(I(x,y)-u(x,y)\right)\right)}^{2}dxdy+\alpha \;\mathrm{Length}\left(C\right).$$

The contour evolution follows a gradient descent scheme:

(29) $$\frac{\partial \phi }{\partial t}=-\frac{\delta E_{\mathrm{Local}\_\mathrm{MS}}}{\delta \phi }.$$

A unified active contour energy model is further adopted:

(30) $$E\left(\phi \right)=\int \left[\alpha |\nabla \phi |+F(\phi ,I)\right]dxdy,$$

where F(ϕ,I) denotes the image-based data term. The local mean intensities inside and outside the contour are computed as

(31) $$c_{1}=\frac{\sum_{\mathrm{inside}}I\left(x\right)}{\left|\mathrm{inside}\right|},$$

(32) $$c_{2}=\frac{\sum_{\mathrm{outside}}I\left(x\right)}{\left|\mathrm{outside}\right|}.$$

The image driven force term is defined as

(33) $$F_{\mathrm{data}}=\nabla \cdot \left[\left(c_{1}^{2}-c_{2}^{2}\right)\delta \left(\phi \right)\right]-2I\nabla \cdot \left[\left(c_{1}-c_{2}\right)\delta \left(\phi \right)\right].$$

The curvature of the contour is approximated as

(34) $$k=\frac{\partial^{2}\phi }{\partial x^{2}}+\frac{\partial^{2}\phi }{\partial y^{2}}.$$

The level-set evolution equation is then written as

(35) $$\frac{\partial \phi }{\partial t}=\delta \left(\phi \right)\left(-F_{\mathrm{data}}+\alpha k\right).$$

To maintain the signed distance property, re-initialization is performed using

(36) $$\frac{\partial \phi }{\partial t}=\frac{\phi -\mathrm{sign}(\phi )}{\sqrt{1+{\left|\nabla \phi \right|}^{2}}}.$$

#### 3.4.4. LIF Energy Model with Gaussian Regularization

To further enhance boundary precision, a LIF energy model is applied to refine the evolving contour. This model leverages local intensity statistics to improve segmentation accuracy, particularly in regions with weak or inhomogeneous boundaries. The LIF energy functional is defined as

(37) $$E_{\mathrm{LIF}}\left(\phi ,f_{1},f_{2}\right)=\int_{\Omega }\left[\phi {\left(I-f_{1}\right)}^{2}+\left(1-H(\phi )\right){\left(I-f_{2}\right)}^{2}\right]dxdy,$$

where *I* denotes the image intensity, H(ϕ) is the Heaviside function, and $f_{1}$ and $f_{2}$ represent the local mean intensities inside and outside the contour, respectively. The level-set evolution equation minimizing the LIF energy is given by

(38) $$\frac{\partial \phi }{\partial t}=\delta \left(\phi \right)\left[-\frac{\partial E_{\mathrm{LIF}}}{\partial \phi }\right],$$

where δ(ϕ) is the Dirac delta function. After each iteration, the level-set function is smoothed using Gaussian convolution to ensure contour regularity:

(39) $$\phi =\phi *K_{\phi },$$

where $K_{\phi }$ is a Gaussian kernel with standard deviation $\sigma_{\phi }$. An extended LIF formulation incorporating intensity bias correction is expressed as

(40) $$E_{\mathrm{LIF}}\left(\phi \right)=\int_{\Omega }\left[\gamma {\left(I_{bc}\left(x,y\right)-\bar{I}\right)}^{2}+\delta \left(\phi \left(x,y\right)\right)\right]dxdy,$$

where $I_{bc}$ denotes the bias corrected image and $\bar{I}$ represents the mean intensity inside the evolving contour. The final refined segmentation mask is obtained as

(41) $$S_{r}\left(x,y\right)=\left\{\begin{array}{cc} 1, & \mathrm{if}\;\phi (x,y)>0,\\ 0, & \mathrm{if}\;\phi (x,y)<0. \end{array}\right.$$

#### 3.4.5. Panoptic-Style Tumor Segmentation

Following semantic segmentation, a panoptic segmentation strategy is employed to distinguish multiple tumor instances within contiguous regions. The refined semantic segmentation mask is defined as

(42) $$S_{r}\left(x,y\right)\in \left\{0,1\right\},\;\left(x,y\right)\in \Omega ,$$

where foreground pixels correspond to tumor tissue. Connected component labeling is applied to identify candidate tumor regions:

(43) $${\left\{C_{i}\right\}}_{i=1}^{N}=\mathrm{CCL}\left(S_{r}\right),$$

where $C_{i}\subset \Omega$ denotes the *i*-th connected tumor component. For each connected region, a Euclidean distance transform is computed as

(44) $$D_{i}\left(x,y\right)=\mathrm{dist}\left(\left(x,y\right),\partial C_{i}\right),\;\left(x,y\right)\in C_{i},$$

where $\partial C_{i}$ represents the boundary of region $C_{i}$. Local maxima of the distance map are extracted as tumor instance centers:

(45) $$P_{i}=\left\{\left(x,y\right)\;|\;D_{i}\left(x,y\right)\;\mathrm{is}\;a\;\mathrm{local}\;\mathrm{maximum}\right\}.$$

Using these markers, marker-controlled watershed segmentation is applied:

(46) $${\left\{L_{i,j}\right\}}_{j=1}^{M_{i}}=\mathrm{Watershed}\left(D_{i},P_{i}\right),$$

where $L_{i,j}$ denotes the *j*-th tumor instance within component $C_{i}$. Each pixel is then assigned both a semantic label and an instance identifier, forming the panoptic segmentation map:

(47) $$P\left(x,y\right)=\left(S_{r}\left(x,y\right),\mathrm{ID}\left(x,y\right)\right),$$

with

(48) $$\mathrm{ID}\left(x,y\right)=\left\{\begin{array}{cc} j, & \mathrm{if}\;\left(x,y\right)\in L_{i,j},\\ 0, & \mathrm{if}\;S_{r}\left(x,y\right)=0. \end{array}\right.$$

To enforce anatomical plausibility, each tumor instance undergoes morphological regularization:

(49) $$L_{i,j}^{*}=\mathrm{Morph}\left(L_{i,j}\right),$$

where Morph(·) includes operations such as hole filling, boundary smoothing, and removal of small regions. Instances failing size constraints are discarded:

(50) $$L_{i,j}^{*}=\left\{\begin{array}{cc} L_{i,j}^{*}, & |L_{i,j}^{*}|\geq \tau ,\\ \emptyset , & \mathrm{otherwise}. \end{array}\right.$$

The final panoptic segmentation output is defined as

(51) $$P^{*}\left(x,y\right)=\left(S_{r}\left(x,y\right),{\mathrm{ID}}^{*}\left(x,y\right)\right).$$

### 3.5. Evaluation Metrics

The experimental results, including the DSC [39] and IoU [40], demonstrate a significant improvement in segmentation, confirming the effectiveness of the refinement process in achieving clinically meaningful outcomes. The DSC is defined as:

(52) $$\mathrm{DSC}=\frac{2|G\cap S|}{\left|G|+|S\right|},$$

where *G* denotes the ground truth and *S* represents the segmented region. The IoU is given by:

(53) $$\mathrm{IoU}=\frac{\left|G\cap S\right|}{\left|G|+|S|-|G\cap S\right|}.$$

The variability of per-image performance is summarized using the interquartile range (IQR), defined as

(54) $$\mathrm{IQR}=Q_{3}-Q_{1},$$

where $Q_{1}$ and $Q_{3}$ are quartiles.

(55) $$t=\frac{\bar{d}}{s_{d}/\sqrt{n}},$$

where $\bar{d}$ is the mean difference, $s_{d}$ is the standard deviation, and *n* is the sample size. For DSC, the *t*-statistic is computed as:

(56) $$t_{\mathrm{DSC}}=\frac{{\bar{X}}_{\mathrm{DSC}}}{SE_{\mathrm{DSC}}},$$

with the corresponding *p*-value:

(57) $$p_{\mathrm{DSC}}=P\left(T\leq t_{\mathrm{DSC}}\right).$$

Similarly, for IoU:

(58) $$t_{\mathrm{IoU}}=\frac{{\bar{X}}_{\mathrm{IoU}}}{SE_{\mathrm{IoU}}},$$

and

(59) $$p_{\mathrm{IoU}}=P\left(T\leq t_{\mathrm{IoU}}\right).$$

To account for multiple comparisons, the Holm–Bonferroni adjusted *p*-values are:

(60) $$p_{\mathrm{DSC}}^{\mathrm{adj}}=\max\;\left(2p_{\mathrm{DSC}},\;p_{\mathrm{IoU}}\right),$$

(61) $$p_{\mathrm{IoU}}^{\mathrm{adj}}=\max\;\left(2p_{\mathrm{IoU}},\;p_{\mathrm{DSC}}\right).$$

The CI for DSC is defined as:

(62) $${\mathrm{CI}}_{\mathrm{DSC}}=\left[{\hat{\mu }}_{\mathrm{DSC}}-z_{\alpha /2}{\hat{\sigma }}_{\mathrm{DSC}},\;{\hat{\mu }}_{\mathrm{DSC}}+z_{\alpha /2}{\hat{\sigma }}_{\mathrm{DSC}}\right],$$

and similarly for IoU:

(63) $${\mathrm{CI}}_{\mathrm{IoU}}=\left[{\hat{\mu }}_{\mathrm{IoU}}-z_{\alpha /2}{\hat{\sigma }}_{\mathrm{IoU}},\;{\hat{\mu }}_{\mathrm{IoU}}+z_{\alpha /2}{\hat{\sigma }}_{\mathrm{IoU}}\right].$$

The Panoptic Quality (PQ) metric is defined as:

(64) PQ=SQ×RQ,

where the Segmentation Quality (SQ) is given by:

(65) $$\mathrm{SQ}=\frac{1}{\left|TP\right|}\sum_{(p,g)\in TP}\mathrm{IoU}\left(p,g\right),$$

and the Recognition Quality (RQ) is defined as:

(66) $$\mathrm{RQ}=\frac{\left|TP\right|}{\left|TP\right|+\frac{1}{2}\left|FP\right|+\frac{1}{2}\left|FN\right|}.$$

This robustness is ascribed to (i) MICO-based bias correction, which ensures uniform input, and (ii) the localized refinement step (LAC–LIF), which tailors contour evolution to local intensity fluctuations. Subsequent efforts will enhance this by integrating automated hyperparameter optimization techniques over more extensive datasets shown in Table 2.

Up to 800 iterations were used for optimal refinement and boundary segmentation, while a minimum of 300 was chosen for processing efficiency and segmentation precision. The trials used 300 to 800 iterations to adjust for speed and precision describe in Table 3.

The complete pipeline necessitates roughly 76 s per image during high precision LAC refining; nevertheless, the proposed system is designed for offline or near-real-time clinical decision assistance, where asynchronous processing durations of several seconds are permissible. The majority of the runtime is attributed to the LAC stage, and diminishing iterations decreases processing time to approximately 26.5 s; more acceleration is anticipated via improved implementations, parallelization, GPU utilization, or ROI processing.

## 4. Experimental Results and Analysis

The proposed hybrid framework for breast cancer identification in mammographic images demonstrates significant improvements in segmentation accuracy, as reflected by key quantitative performance metrics. The integration of advanced preprocessing and refinement stages effectively addresses intensity inhomogeneities, noise artifacts, and ambiguous boundary definitions, resulting in more accurate delineation of suspicious regions. Deep-learning-related methods are included as reference benchmarks to provide contextual comparison with commonly adopted data driven approaches. The purpose of this comparison is not to claim direct superiority over highly optimized deep architectures, but rather to emphasize the complementary strengths of the proposed framework, particularly with respect to interpretability, robustness, and clinically motivated behavior. It is important to note that deep learning approaches typically depend on large scale annotated datasets, extensive training procedures, and GPU acceleration. In contrast, the proposed framework is training free and mathematically driven, enabling reliable performance and enhanced interpretability in limited data and resource-constrained scenarios.

### 4.1. Quantitative Evaluation of Semantic Segmentation on MIAS Dataset

The initial segmentation using Multiplicative Intrinsic Component Optimization MICO_2D provided a bias-corrected foundation, yielding a DSC of 0.9230 and an IoU of 0.8569. While this initial segmentation was effective in addressing intensity variations, further enhancements were necessary for precise boundary detection. The intensity inhomogeneity correction method based on a clustering criterion further standardized contrast levels, optimizing segmentation accuracy. Although the dataset used in this study is not newly released, it remains a clinically validated benchmark that is still widely employed in recent mammographic segmentation studies, particularly for dense breast analysis. Public datasets with pixel-level expert annotations for dense mammograms are limited, and the selected dataset continues to provide a realistic and challenging evaluation environment.

The globalized segmentation step, leveraging an improved Vese–Chan multiphase level-set model with a DR term, significantly enhanced boundary delineation and segmentation robustness. This approach led to improved convergence and ensured smooth level-set evolution, reducing the impact of noise and intensity irregularities. However, some regions still exhibited segmentation ambiguity, necessitating further refinement.

**Figure 2:**
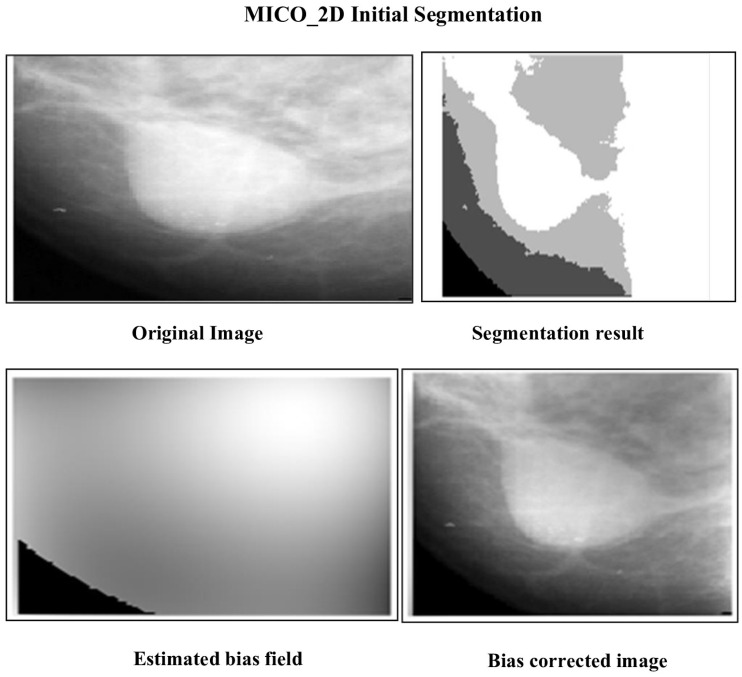
Segmentation results of mammographic images using MICO_2D.

**Figure 3:**
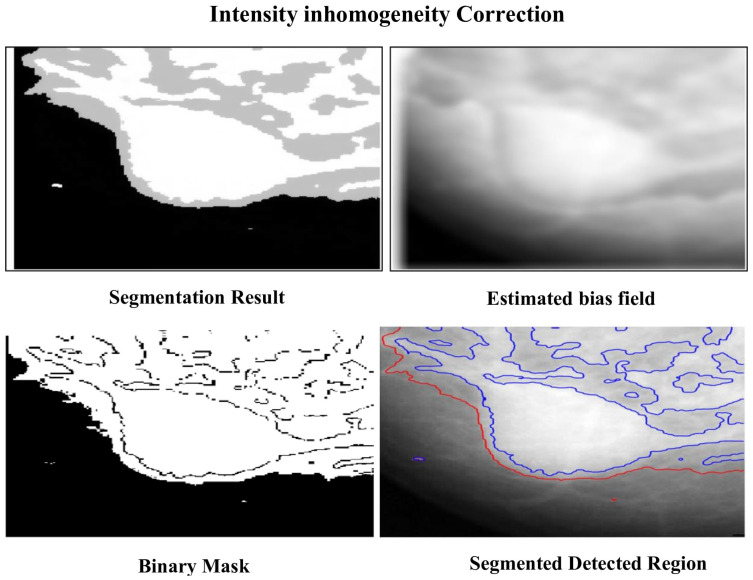
Segmentation results using method intensity inhomogeneity correction.

**Figure 4:**
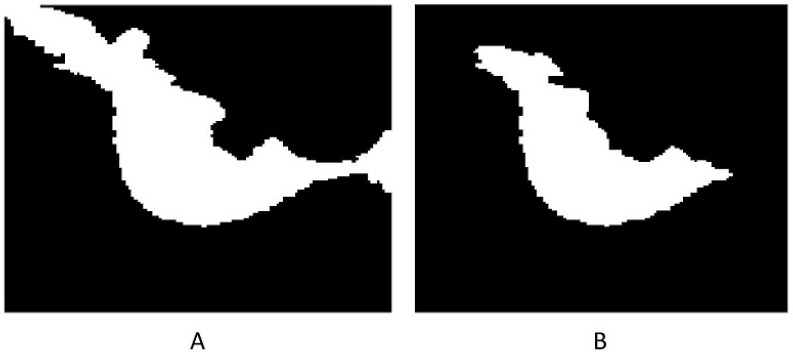
LAC segmentation results on mammographic images: (**A**) Lesion segmentation by ACMS, and (**B**) lesion segmentation by ACUM segmentation.

The methods progressively improve the accuracy and quality of segmentation, addressing various challenges such as intensity inhomogeneities, boundary delineation, and noise artifacts. The segmentation results of the proposed methods are presented in Figure 2 and Figure 3, which show the original mammogram image alongside the segmentation outcomes for each method. The segmentation based on MICO_2D provides an initial bias-corrected result, addressing intensity inhomogeneities but requiring further refinement in complex tissue regions. Intensity inhomogeneity correction enhances contrast and standardizes intensity levels, though some artifacts persist. The Vese-Chan multiphase level-set model with a DR term improves boundary delineation and segmentation accuracy by reducing noise and handling intensity variations. Localized refinement using the LAC and LIF models with Gaussian filtering further sharpen boundaries and enhance segmentation quality, particularly in ambiguous regions. The ACMS model effectively handles intensity inhomogeneity and noise, while the ACUM method improves segmentation by minimizing localized energy fluctuations, aiding in early breast cancer detection. The LIF energy model refines segmentation by aligning local intensity distributions and reducing noise, making it highly effective for detecting subtle features. These advanced techniques collectively enhance segmentation accuracy and efficiency, significantly improving breast cancer detection in mammography. Figure 4 illustrated the LAC results images.

**Figure 5:**
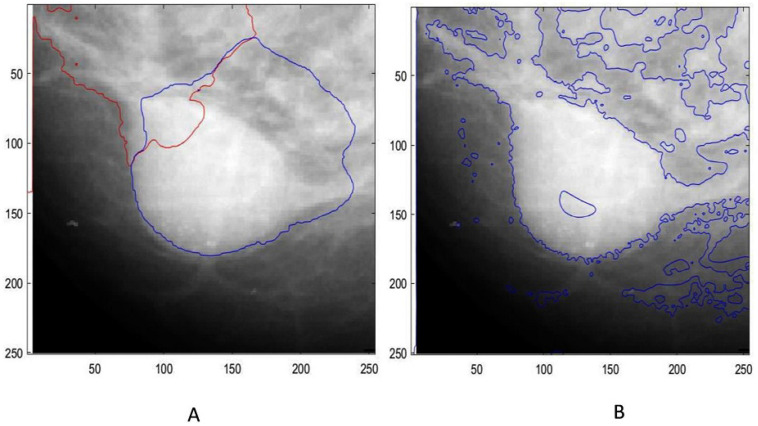
(**A**) Vese–Chan model, and (**B**) LIF energy model with Gaussian filtering (lesion detection).

The localized refinement process, incorporating the LAC model and LIF energy model with Gaussian filtering regularization, successfully refined the contours in challenging regions illustrated in Figure 5 and also vese-chan model. This two-stage refinement approach substantially improved segmentation precision, increasing the DSC to 0.9542 and IoU to 0.9124. The statistical significance of these improvements was confirmed through a t-statistic of −25.1316 (*p* = 1.4883 × 10^−5^) for DSC and −46.0902 (*p* = 1.3254 × 10^−6^) for IoU. Additionally, CI for the improvements in DSC (0.02734–0.03148) and IoU (0.0538–0.0578) validated the robustness of the proposed framework.

**Figure 6:**
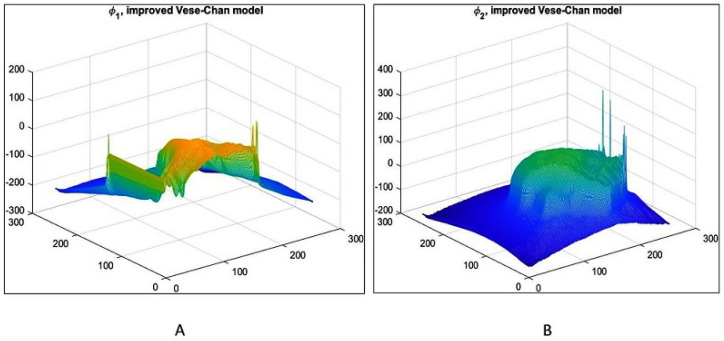
(**A**) 3D surface visualization of the level-set function $\phi_{1}$, illustrating segmentation refinement and regions influenced by noise suppression. (**B**) 3D surface of $\phi_{2}$ obtained from the distance-regularized Vese-Chan model, highlighting stable ROI formation and smooth boundary evolution in dense mammographic tissue.

The proposed model demonstrates strong lesion segmentation capability, as illustrated in Figure 6. The 3D surface plot of the level-set function $\phi^{2}$ shows smooth evolution under DR, which promotes stable boundary propagation and suppresses spurious oscillations in noisy regions. This behavior is essential for accurate tumor delineation in dense mammograms. The gradual elevation of the surface corresponds to the segmented region of interest, while minor surface irregularities indicate areas where additional preprocessing or noise suppression could further improve performance.

**Figure 7:**
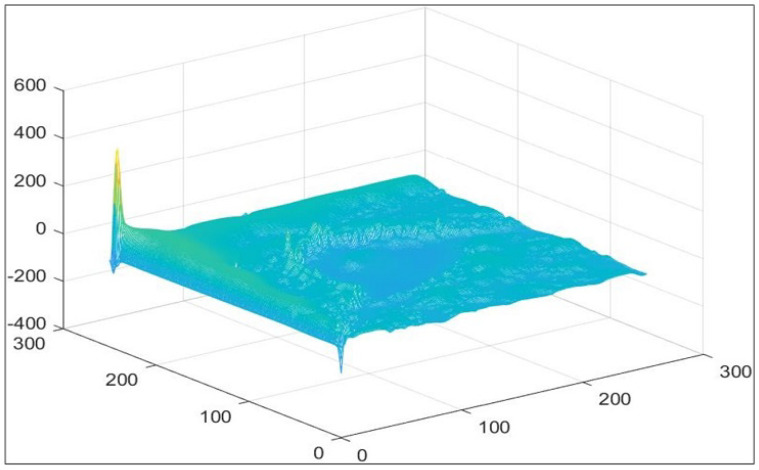
Localized refinement using the LIF energy model with Gaussian filtering. The 3D surface plot of the level-set function ϕ(x,y) after 600 iterations demonstrates refined tumor boundaries, improved local contrast adaptation, and enhanced structural continuity in dense breast regions.

Table 4 presents representative per-image segmentation results for selected MIAS mammograms. The table reports DSC and IoU values for both initial and refined segmentations, along with delta values indicating the change due to refinement. Mean ± standard deviation (SD) and median (IQR) are provided to highlight central tendency and variability. The results show that the refinement stage generally improves segmentation accuracy, particularly for images with moderate tissue density, while maintaining reasonable performance for challenging cases. Most images benefit from refinement; however, those with low contrast, high noise, or complex tumor morphology sometimes exhibit minor reductions in DSC and IoU, reflecting natural variability. It should be noted that comparisons between initial and refined outputs of the same pipeline are illustrative and do not constitute independent validation, as large differences or t-values can be artificially inflated due to dependency between these outputs. These illustrative results complement the full per-image evaluation of all images, which provides robust and statistically independent validation of the proposed framework. Figure 7 presents the 3D surface visualization of the LIF energy model with Gaussian filtering after 600 iterations, demonstrating refined boundary localization and improved adaptation to local intensity variations within dense breast tissue.

**Figure 8:**
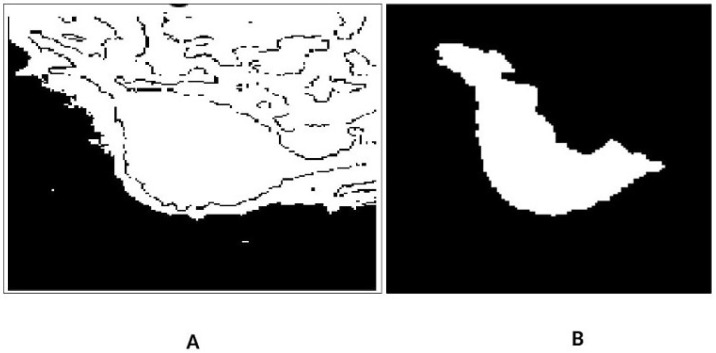
Illustrates the segmentation refinement process. The initial segmentation (**A**) exhibits over-segmentation, where surrounding normal tissues are incorrectly classified as lesion regions, leading to a higher false positive rate. The refined segmentation (**B**), obtained using ACMS, demonstrates improved boundary delineation and effective suppression of non-lesion regions. This refinement enhances segmentation accuracy and reduces false positives, resulting in a more precise lesion representation.

These observations emphasize the robustness of the model and suggest potential improvements for enhanced segmentation accuracy. The 3D surface representation visually demonstrates the segmentation process, with the smooth surface elevation corresponding to the ROI and well defined boundaries distinguishing the object from the background. The DR term’s role in regularization is crucial for preventing overfitting and ensuring smooth, stable boundary delineation, especially in regions with noise or intensity variations. Overall, the 3D surface serves as a valuable tool for visualizing and validating the segmentation model’s effectiveness. The refined segmentation framework as Figure 8 demonstrates a statistically significant improvement over the initial segmentation, achieving a Dice coefficient increase of 3.12% and an IoU improvement of 5.55%. Fusion-based architectures further enhance segmentation accuracy, with the combined multi-view and multi scale model achieving the highest performance. Ablation analysis confirms the contribution of each component to the final segmentation accuracy. The initial segmentation had a DSC of 0.9230 and IoU of 0.8569, while the enhanced segmentation achieved a DSC of 0.9542 and IoU of 0.9124, showing improvements of 0.0312 and 0.0555, respectively. Table 5 compares the DSC and IoU values for the original and enhanced segmentation methods.

**Figure 9:**
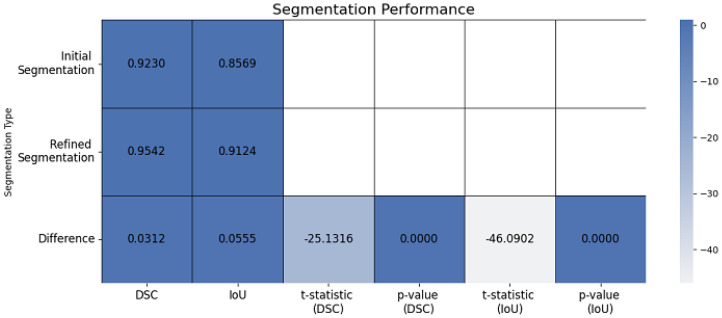
Segmentation performance metrics (DSC and IoU), comparing initial and refined segmentation results.

**Figure 10:**
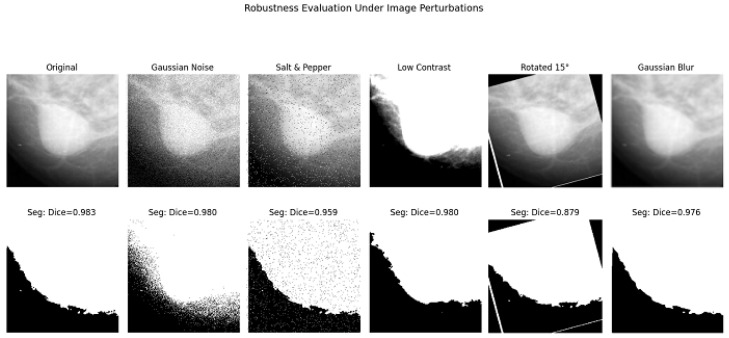
Segmentation results under different image perturbations for a representative test sample.

The segmentation performance was statistically assessed before and after refinement across the dataset using DSC and IoU measures. This comparison underscores the refinement stage’s importance to overall correctness. Table 6 presents a descriptive comparison of initial and refined segmentation results across the dataset using DSC and IoU metrics. The initial segmentation achieved a median DSC of 0.812 [IQR: 0.78–0.84] and a median IoU of 0.684 [IQR: 0.65–0.72]. After refinement, these metrics increased to 0.841 [0.82–0.87] for DSC and 0.738 [0.71–0.77], corresponding to mean improvements of +0.029 and +0.054, respectively. Bootstrap confidence intervals further confirm that these improvements were consistently observed across images ([0.027, 0.031] for DSC and [0.054, 0.058] for IoU). Because the refinement stage is a deterministic post-processing step applied to the same initial segmentations, the paired per-image *t*-statistics reported in Table 7 and Figure 9 are presented strictly as measures of descriptive consistency rather than independent inferential validation. The large absolute *t*-values (DSC: t=−25.13, IoU: t=−46.09) primarily reflect the systematic direction and stability of performance gains across cases. To further evaluate the robustness of the proposed framework (Table 8 and Figure 10), a robustness analysis was performed using a representative test image to elucidate the sensitivity of the segmentation pipeline under controlled perturbations. The image underwent typical distortions, such as Gaussian noise, salt and pepper noise, contrast diminution, a $15^{\circ }$ rotation, and Gaussian blur. This analysis was conducted on a singular representative image for demonstrative purposes.

**Figure 11:**
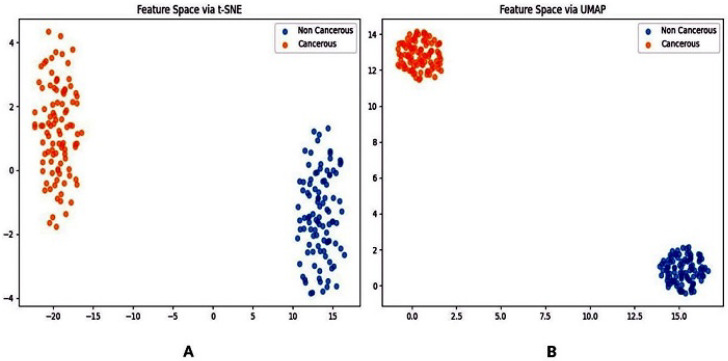
(**A**) t-SNE and (**B**) UMAP visualization of learned mammography feature embeddings, showing clear clustering and separability across breast tissue categories.

In addition, Figure 11 presents visualizations of learned feature embeddings using t-SNE and UMAP, further supporting the discriminative capability of the proposed framework.

The proposed hybrid framework achieves competitive performance relative to deep learning–based baselines while maintaining computational efficiency and methodological transparency in mammographic tumor segmentation. The observed improvements in segmentation accuracy indicate strong potential for clinical applicability, particularly for early breast cancer detection in dense breast tissue. Robust performance across multiple evaluation metrics demonstrates the effectiveness of the refined segmentation stage. High sensitivity reflects accurate identification of true tumor regions, while high precision indicates effective suppression of false positives. The balanced F1-score further confirms consistency between precision and recall. Strong spatial agreement between predicted and reference masks, as reflected by DSC and IoU, indicates reliable delineation of tumor boundaries in challenging dense mammograms.

**Figure 12:**
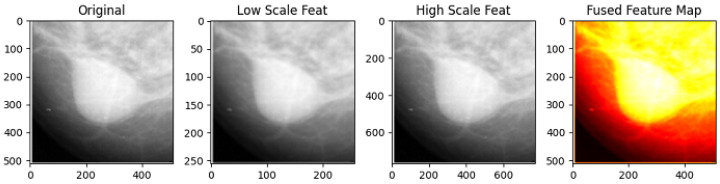
Multi-scale feature extraction and fusion process. Low-, original, and high-resolution inputs are processed and fused to enhance dense tissue representation in mammograms.

**Figure 13:**
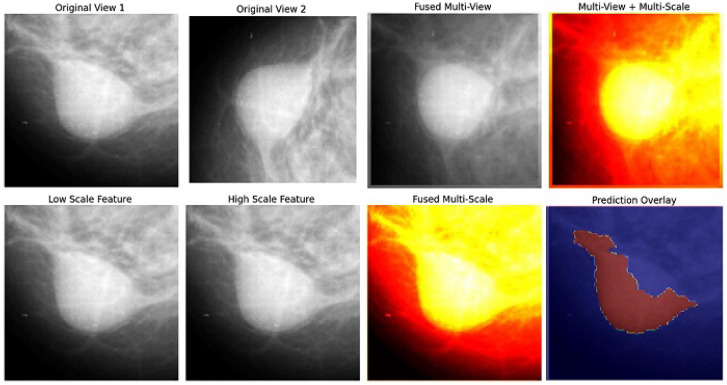
Benchmark multi-scale and multi-view fusion model for comparative evaluation. **Top**: original views and their fused outputs. **Bottom**: low/high-scale features, their fusion, and predicted segmentation overlay. Used as a deep learning baseline against the proposed traditional framework.

Figure 12 and Table 9 illustrate the multi-scale feature extraction and fusion strategy used in the comparative deep learning framework. The mammographic images are processed at multiple spatial resolutions to capture complementary structural information, followed by feature fusion to enhance tissue boundary representation. Figure 13 further visualizes the benchmark multi-scale and multi-view fusion model, showing CC and MLO views, their fused representations, and the corresponding predicted segmentation overlays. While deep learning models often achieve strong performance when trained on large-scale annotated datasets, they typically require extensive data and careful hyperparameter tuning, and may exhibit limited interpretability. In contrast, the proposed hybrid framework is training-free and relies on explicit mathematical modeling, enabling transparent behavior and stable performance under perturbations. To ensure a fair and controlled comparison, all deep learning baselines were evaluated on the same MIAS images and ground-truth masks, without additional external data or dataset augmentation. The comparative analysis is therefore intended to highlight methodological differences and robustness characteristics under identical data availability rather than to establish absolute dominance in segmentation accuracy.

Table 10 displays the findings of the ablation study, demonstrating the impact of each component on segmentation performance. The Vese–Chan model provides a solid baseline for dense tissue segmentation, demonstrating reasonable spatial overlap between predicted and reference regions. The integration of the MICO_2D component yields a modest improvement by addressing intensity inhomogeneity, while the incorporation of LAC leads to a substantial enhancement in segmentation quality through improved boundary localization. Among the individual components, the LIF module contributes the most significant refinement by effectively capturing local intensity variations. The combination of all components in the full refined segmentation model results in the strongest overall performance, highlighting the cumulative contribution of each module and underscoring the critical role of LIF in achieving precise boundary delineation. For comparison, a deep-learning-based fusion architecture also demonstrates strong segmentation capability. However, the proposed hybrid framework offers improved interpretability and consistency, which are essential for clinical applicability. However, when compared with the proposed hybrid traditional framework that integrates MICO_2D, enhanced multiphase level-set segmentation, and localized refinement using LAC and LIF, the proposed method demonstrates superior accuracy and robustness, particularly in handling intensity inhomogeneities and ambiguous boundaries. Although the proposed framework demonstrated strong performance on breast cancer mammogram images, certain limitations should be acknowledged. The assertion of clinical interpretability is presently substantiated by visual analyses, including segmentation overlays and feature embedding visualizations (t-SNE and UMAP). A formal user study with radiologists was not performed, which constrains direct inferences regarding clinical usability. The approach may exhibit diminished performance in difficult scenarios, such as significant rotations or very low contrast mammograms, as indicated by the robustness trials. Although mammograms are generally obtained with appropriate alignment in clinical settings, these instances underscore the necessity for improved rotation-invariant methodologies. Ultimately, although the results from public datasets are promising, the relatively small sample sizes may limit their applicability to wider clinical populations. Future endeavors will rectify these limitations by integrating rotation invariant mechanisms (e.g., sophisticated data augmentation or spatial transformer networks), broadening validation across extensive multi-center datasets, and performing radiologist-in-the-loop evaluations to thoroughly evaluate clinical applicability.

### 4.2. Validation on INBreast Dataset

To further assess generalizability and robustness beyond the scanned-film MIAS dataset, the proposed framework was evaluated on the INbreast dataset. This publicly available collection consists of 410 full-field digital mammograms (high-resolution, 14-bit depth) from 115 cases, featuring precise pixel-level annotations for masses, calcifications, and asymmetries, with a notable proportion of dense breasts and complex morphologies. Absolute segmentation metrics on INbreast are comparable with MIAS, benefiting from digital acquisition and reduced artifacts, though challenged by higher resolution and tissue complexity. The refinement stage consistently delivers meaningful improvements. This cross-dataset validation confirms the method’s ability to handle more modern, clinically representative digital mammograms without retraining or parameter retuning.

**Figure 14:**
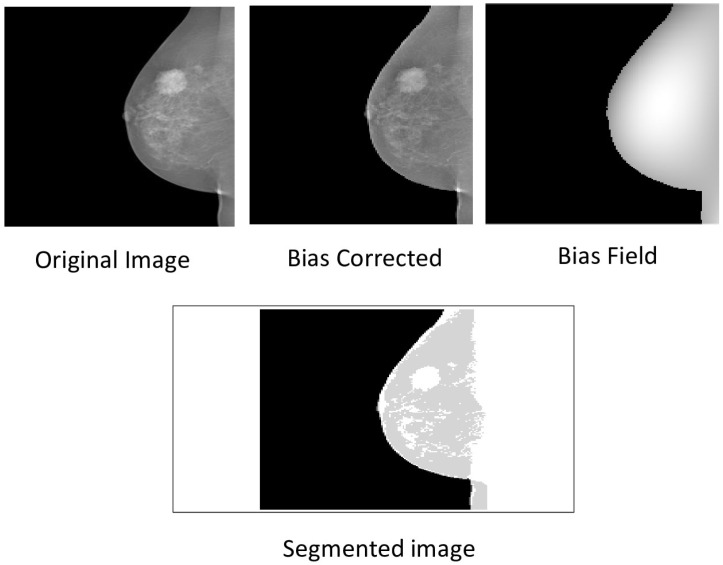
Segmentation results of mammographic images using MICO_2D on INBreast Dataset.

Figure 14 illustrates segmentation outcomes using MICO_2D on representative INbreast images. The original mammogram exhibits significant low-frequency intensity inhomogeneity across the breast parenchyma, which can degrade lesion visibility and boundary accuracy. The estimated bias field captures these gradual variations, and post correction yields a more uniform intensity distribution, enhancing tissue contrast and enabling precise tumor demarcation in the segmented output. This validates the effectiveness of MICO_2D in addressing acquisition-induced bias in digital mammograms.

**Figure 15:**
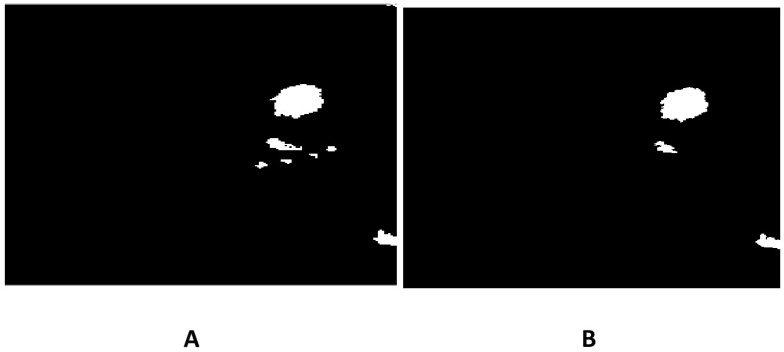
(**A**) Initial segmentation. (**B**) Refined segmentation on the INBreast Dataset.

**Figure 16:**
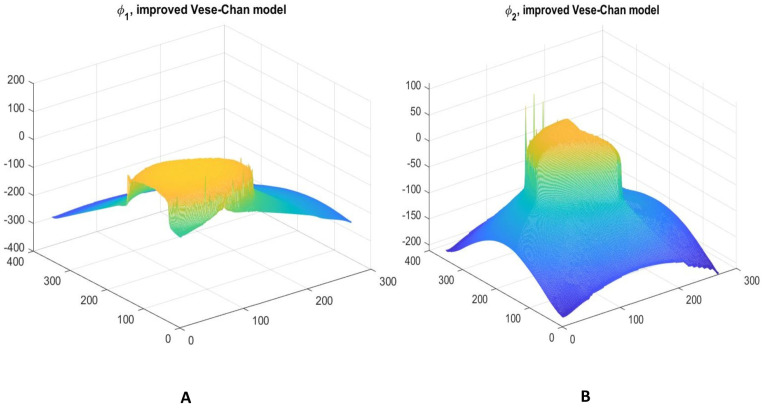
3D surface representation of the level-set evolution on the INbreast dataset. (**A**) Initial level-set function $\phi_{1}$ showing preliminary contour placement with some irregularities. (**B**) $\phi_{2}$ from the distance-regularized multiphase Chan-Vese model exhibiting smoother topology and better edge adherence.

Figure 15 compares initial (post-global) and refined segmentations: (A) initial output shows reasonable coarse tumor capture but residual over segmentation or leakage in ambiguous regions; (B) refined output demonstrates sharpened, anatomically plausible boundaries with reduced false positives, highlighting the value of localized LAC-LIF refinement. Figure 16 further elucidates the refinement process on INbreast. (A) Illustrates the three dimensional surface representation of the initial level-set function $\phi_{1}$, showing the contour initialization and its spatial distribution throughout the image domain. The surface topology reflects the preliminary segmentation state prior to refinement. (B) Presents the 3D surface of $\phi_{2}$, obtained using the distance-regularized Chan-Vese model. In comparison to $\phi_{1}$, the refined level-set function demonstrates smoother transitions and more stable contour evolution, indicating improved boundary adherence to the tumor region. The distance regularization term ensures numerical stability and prevents irregular contour deformation during the segmentation process.

Quantitative evaluation demonstrates that the proposed hierarchical pipeline effectively mitigates the limitations of early-stage segmentation and progressively converges toward clinically reliable tumor delineation. Table 11 reveals a substantial overall improvement following localized refinement, with mean DSC rising from 0.904 to 0.932 and IoU from 0.825 to 0.884. These gains of approximately 3.1% in DSC and 7.2% in IoU indicate that the two-stage LAC-LIF process significantly enhances spatial overlap with expert annotations, particularly in regions of weak gradient, subtle intensity transitions, or complex dense tissue patterns characteristic of digital mammograms. The consistent increase across both overlap metrics confirms the framework’s ability to recover fine boundary details that are critical for accurate tumor volume estimation and margin assessment in clinical decision support, even under the more demanding conditions of the INbreast dataset.

Table 12 provides a distribution-aware perspective on segmentation performance. It shows that median DSC improves from 0.789 to 0.815 and median IoU from 0.679 to 0.731 after localized refinement, with tight bootstrap confidence intervals around the mean differences of +0.026 (DSC) and +0.052 (IoU). The narrow confidence intervals and consistent upward shift in central tendency and quartiles across the dataset demonstrate that the refinement stage delivers reliable, dataset-wide enhancement rather than isolated improvements in easier cases. This robustness in the presence of INbreast’s variability-dense parenchyma, subtle lesion margins, and complex intensity transitions-highlights the adaptive capability of the localized active contour and LIF models to effectively address patient-specific morphological and intensity challenges in digital mammograms.

Table 13 illustrates case-level behavior on representative images. Positive delta values in nearly all instances demonstrate that the refinement stage systematically corrects initial under- or over-segmentation, particularly in moderately to highly challenging cases (e.g., IDs 3 and 4). The positive mean delta (≈0.037 for DSC) and narrow standard deviation indicate that the framework provides predictable boundary enhancement even in the presence of substantial tissue complexity, reinforcing its suitability as a decision-support tool where consistency across patients is essential.

Table 14 reports paired per-image t-statistics for the differences between initial and refined segmentation outputs. The t-values and small *p*-values reflect the consistent directional nature of the performance improvement across images. These results should be interpreted as descriptive evidence of systematic refinement benefit within the dependent pipeline, rather than as independent inferential validation against a null hypothesis of no difference.

Comparative evaluation against multi-scale and multi-view deep learning baselines on the same INbreast subset (Table 15) shows strong performance by fusion models, with the multi-view + multi-scale variant reaching DSC 96.53% and IoU 93.51%. However, the proposed hybrid framework delivers competitive accuracy (DSC 0.932, IoU 0.884) with superior transparency and no training data requirement.

Table 16 elucidates the synergistic contribution of each module. Starting from a solid bias-corrected baseline MICO_2D, the distance-regularized Vese–Chan model provides global structural coherence, while LAC substantially improves localization. The largest single-step gain occurs with LIF, which leverages local intensity statistics to resolve ambiguous boundaries. The cumulative progression to 0.932 DSC demonstrates that the hybrid design effectively decomposes the segmentation problem into complementary stages—each addressing a distinct challenge (bias, global separation, and local precision)—resulting in superior final delineation compared to any individual component.

Robustness under perturbations remains strong (Dice > 94% for noise/blur/low-contrast; minor drop to ∼86.5% at 15° rotation), consistent with MIAS findings. Comparative evaluation against multi-scale and multi-view deep learning baselines on the same INbreast subset shows strong performance by fusion models, with the multi-view + multi-scale variant reaching DSC 96.53% and IoU 93.51%. However, the proposed hybrid framework delivers competitive accuracy (DSC 0.932, IoU 0.884) with superior transparency, deterministic behavior, and no requirement for large annotated training data—qualities particularly valuable in clinical decision support and limited-data scenarios.

### 4.3. Rotational Robustness

This framework evaluated the robustness of the proposed system under in-plane rotations (0°, ±15°, ±30°) by jointly rotating the mammograms and corresponding ground truth masks using consistent interpolation. The findings demonstrate a consistent decline in segmentation accuracy with increasing rotation angles, signifying sensitivity to orientation variations. This behavior is anticipated, as the proposed framework depends on localized intensity statistics, directional gradients, and contour evolution within spatial neighborhoods that are intrinsically orientation-dependent. No explicit spatial normalization or rotation invariant preprocessing was used, as the methodology was intended to function directly on anatomically preserved mammographic images (CC and MLO), where significant rotational aberrations are rare in standard clinical acquisition. The observed performance decline underscores a restriction of the existing formulation, while also indicating a conscious design decision to maintain anatomical accuracy. Integrating lightweight spatial normalization or rotation-invariant neighborhood formulations signifies a possible avenue for future enhancements of the system.

### 4.4. Panoptic-Style Tumor Instance Segmentation

**Figure 17:**
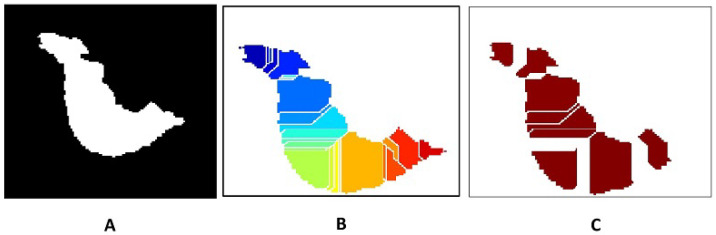
Panoptic-style tumor instance segmentation results. (**A**) Refined semantic segmentation. (**B**) Raw instance separation using marker-controlled watershed. (**C**) Refined panoptic-style segmentation after instance merging, showing 9 distinct tumor instances.

While the preceding sections focus on accurate semantic delineation of tumor regions, clinical interpretation of dense mammograms often requires identifying individual tumor foci within connected or irregularly shaped lesions. To address this requirement, a panoptic-style tumor instance segmentation stage is introduced as a final post-processing extension of the proposed framework. In this study, panoptic segmentation refers to a unified representation in which each pixel is assigned both a semantic tumor label and a unique instance identifier. Although the term is commonly associated with deep-learning-based architectures, the proposed framework achieves a panoptic-style output using deterministic morphological operations, without reliance on learning-related models. After refined semantic segmentation, the binary tumor mask is transformed using a Euclidean distance transform, where local maxima correspond to tumor core regions. These maxima are used as internal markers to initialize instance separation. Marker-controlled watershed segmentation is then applied on the negative distance map to separate connected tumor regions while preserving boundary integrity. Each resulting region is assigned a unique instance label, producing a panoptic representation P(x,y) that encodes both semantic class and instance identity. Representative results are illustrated in Figure 17. Figure 17A shows the refined semantic segmentation, Figure 17B presents the raw instance separation obtained via watershed, and Figure 17C shows the refined panoptic result after instance merging. This merging step reduces over-segmentation by consolidating 27 raw tumor regions into 9 clinically meaningful tumor instances, enabling improved structural interpretation in dense breast tissue.

Quantitative results of the panoptic-style segmentation stage are summarized in Table 17. Perfect pixel-level agreement (Dice and IoU of 1.000) is observed by design, as the panoptic representation is derived directly from the refined semantic mask. At the instance level, all detected tumor regions are preserved without loss or fragmentation. Panoptic evaluation further reports a SQ of 0.8451 and a RQ of 1.000, resulting in a PQ of 0.8451.

It should be noted that the reported panoptic metrics do not represent an independent instance-level evaluation, as the panoptic extension is a deterministic post-processing step based on morphological and topological cues. True instance-level validation would require datasets with explicit tumor instance annotations, which are currently unavailable for dense mammography. Future work will focus on validating this panoptic extension using such datasets and through expert clinical assessment. Overall, the proposed panoptic extension enhances interpretability by enabling the separation and visualization of individual tumor instances, supporting more detailed structural analysis while preserving the pixel-level accuracy of the underlying semantic segmentation. Since the panoptic representation is obtained as a deterministic post-processing step from the refined semantic mask, pixel-level Dice and IoU scores remain identical by design and do not constitute an independent segmentation evaluation. Therefore, instance level and panoptic quality metrics provide a more appropriate assessment of this stage.

## 5. Limitations and Scope of the Proposed Framework

The proposed framework demonstrates strong and consistent performance in dense mammography segmentation; however, several limitations should be acknowledged. The primary evaluation was performed on the MIAS dataset, which may limit generalizability across imaging devices, acquisition protocols, and patient populations. Evaluation on the INBreast dataset showed consistent improvements after refinement, though absolute performance remains lower due to higher resolution and more complex tissue structures. Instance-level ground truth annotations are not available in MIAS or INBreast, so the instance decomposition stage is assessed as a deterministic post-processing extension of refined semantic segmentation rather than independently verified. The framework has not yet been validated in radiologist-in-the-loop studies or multi-center clinical trials, and claims regarding clinical utility are therefore limited. Parameter settings are deterministic and empirically chosen for reproducibility, but their optimality across diverse scanners, breast densities, and imaging conditions has not been fully explored. Finally, deep learning baselines are included as contextual references with standard training configurations, as the focus of this study is a transparent, training-free segmentation framework suitable for small datasets.

These limitations underscore avenues for future research, such as multi-dataset validation, instance-level annotation investigations, radiologist-assisted assessments and a more extensive examination of parameter robustness across various imaging contexts.

## 6. Conclusions

This study introduces a clinically oriented, training-free hybrid segmentation framework designed to deliver robust and interpretable delineation of breast tumors in dense mammograms. The proposed pipeline systematically integrates three complementary optimization-based stages: (1) MICO_2D-based intensity inhomogeneity correction to normalize acquisition artifacts, (2) a distance-regularized multiphase Vese–Chan level-set model for reliable global tumor localization, and (3) a two-stage localized refinement strategy combining localized active contours (LAC) with Local Image Fitting (LIF) energy and Gaussian regularization to achieve precise, smooth boundary delineation in regions of low contrast and tissue heterogeneity. A deterministic panoptic-style instance segmentation module decomposes connected tumor regions into anatomically distinct instances, providing enhanced structural interpretability—such as tumor multiplicity and spatial organization—without altering pixel-level boundaries or conventional overlap metrics. Extensive quantitative evaluation on the MIAS and INbreast datasets demonstrates that the framework achieves strong performance. These results reflect consistent improvements of 3–5.5% across overlap metrics following refinement, supported by statistically significant gains, tight confidence intervals, and ablation studies confirming the synergistic contribution of each component. Despite its strengths, evaluation remains limited to the MIAS and INbreast datasets, with formal clinical validation via multi-center studies or radiologist assessments still pending. The framework shows expected sensitivity to large in-plane rotations, reflecting its reliance on orientation-sensitive features and the deliberate preservation of standardized mammographic views (CC/MLO). Future work will extend validation to larger multi-institutional cohorts, incorporate rotation-invariant techniques, and perform radiologist-in-the-loop evaluations to further establish clinical utility.   

## Figures and Tables

**Table 1:** Performance comparison of various methods for breast cancer detection using mammogram datasets.

Methods	Datasets	Acc.	Sens.	F1-Score	Pr
XGBoost24, [28]	MIAS, CBIS-DDSM	79.81, 78.30	79.13, 78.08	73.68, 70.58	68.93, 64.39
KNN29, [29]	MIAS, CBIS-DDSM	73.91, 74.55	73.91, 74.18	66.92, 66.03	61.15, 59.49
Multiple pre-trained CNN, [30]	RSNA, MIAS, DDSM	92.00, 94.50, 96.00	96.00, 96.32, 94.70	-	92.00, 91.80, 97.00
ACA-ATRUNet-MDN40, [31]	MIAS, CBIS-DDSM	78.88, 79.85	80.00, 79.90	73.016, 72.55	67.15, 66.44
CNN25, [32]	MIAS, CBIS-DDSM	80.12, 79.81	79.13, 79.58	73.98, 72.44	69.46, 66.47
LQP, SVM, [33]	MIAS	94.00	-	-	-
GMM, SVM, [34]	Mini-MIAS	92.50	-	-	-
KNN, [35]	Mini-MIAS	92.00	-	-	-
SVM, Hough, [36]	InBreast	86.13	80.67	-	92.81
Voting Classifier, [37]	MIAS	85.00	-	-	-
CNN-4d, [38]	Mini-MIAS	89.05	90.63	-	83.67
CNN, [39]	DDSM	93.50	-	-	-
CNNs, [40]	DDSM	85.82	82.28	-	86.59
MML-EOO-ACA-ATRUNet-MDN, [20]	MIAS, CBIS-DDSM	89.13, 89.06	88.69, 88.93	85.35, 84.42	82.25, 80.34

**Table 2:** Parameter settings of the proposed hybrid segmentation framework.

Component	Parameter	Value	Description
MICO (Bias Correction)	iterNum	20	Iterations for bias field optimization
	N_region	3	Number of tissue classes
	q	1	Fuzziness exponent (crisp segmentation)
Multiphase Chan–Vese	delta_t	0.1	Time step for level-set evolution
	$\lambda_{1},\lambda_{2}$	1	Region fitting weights
	ν	$0.001\times 255^{2}$	Contour length regularization
	ϵ	1	Heaviside smoothing parameter
LIF	num_it	800–1500	Iterations for local refinement
	rad	5–9	Radius of local fitting window
	α	0.001–0.3	Length regularization weight
Gaussian Regularization	σ	4	Gaussian kernel scale for boundary smoothing
General Settings	ROI threshold	I>5	Background noise suppression

**Table 3:** Stage-wise Runtime Comparison of the Hybrid Segmentation Framework.

Stage	Runtime (800 Iterations)	Runtime (300 Iterations)
MICO (Bias Correction)	1.2454 s	0.7789 s
Vese–Chan	2.8860 s	2.0047 s
LAC	72.8355 s	22.8612 s
Total (Full Pipeline)	76.0857 s	26.5262 s

**Table 4:** Illustrative segmentation results for a small set of MIAS images showing the effect of localized refinement. Mean ± SD and median (IQR) are reported for these representative cases.

Image ID	DSC Initial	DSC Refined	Delta DSC	IoU Initial	IoU Refined	Delta IoU
1	0.947	0.943	−0.005	0.900	0.891	−0.008
2	0.936	0.932	−0.004	0.879	0.873	−0.007
3	0.764	0.717	−0.047	0.618	0.558	−0.059
4	0.561	0.465	−0.096	0.390	0.303	−0.087
Mean ± SD	0.802 ± 0.181	0.764 ± 0.225	−0.038 ± 0.044	0.697 ± 0.242	0.656 ± 0.281	−0.040 ± 0.040
Median (IQR)	0.851 (0.561–0.947)	0.825 (0.465–0.943)	−0.039 (−0.096–(−0.004))	0.749 (0.390–0.900)	0.716 (0.303–0.891)	−0.058 (−0.087–(−0.008))

**Table 5:** Performance comparison between initial and refined semantic segmentation using DSC and IoU.

Segmentation Type	DSC	IoU
Initial Segmentation	0.9230	0.8569
Refined Segmentation	0.9542	0.9124
Difference	0.0312	0.0555

**Table 6:** Comparison of initial and refined segmentation results (median [IQR], mean difference, bootstrap CI).

Metric	Initial (Median [IQR])	Refined (Median [IQR])	Mean Difference	Bootstrap CI (Difference)
DSC	0.812 [0.78–0.84]	0.841 [0.82–0.87]	+0.029	[0.027, 0.031]
IoU	0.684 [0.65–0.72]	0.738 [0.71–0.77]	+0.054	[0.054, 0.058]

**Table 7:** Paired per-image *t*-statistics assessing the statistical significance of differences between initial and refined segmentation.

Metric	t-Value	*p*-Value	Holm-Bonferroni Adjusted *p*
DSC	−25.1316	$1.49\times 10^{-5}$	$1.49\times 10^{-5}$
IoU	−46.0902	$1.33\times 10^{-6}$	$2.65\times 10^{-6}$

Due to the dependent nature of the paired observations (refined outputs are deterministically derived from the initial outputs within the same pipeline), these t-statistics and *p*-values should be interpreted solely as descriptive measures of consistent directional change and magnitude of improvement, rather than as classical independent-sample hypothesis tests. They quantify the systematic stability of refinement benefits across cases.

**Table 8:** Illustrative Robustness Analysis Under Image Perturbations.

Evaluation Scenario	Dice Score (%)
Original	98.28
Gaussian Noise	98.00
Salt & Pepper	95.82
Low Contrast	97.95
Rotated 15°	87.93
Gaussian Blur	97.63

**Table 9:** Performance comparison of multi-scale and multi-view fusion architectures for dense tissue segmentation.

Architecture	Dice (%)	IoU (%)	Sensitivity (%)
U-Net	96.35	91.84	95.12
Multi-Scale Fusion	98.21	94.91	97.76
Multi-View Fusion	98.34	95.03	98.01
Multi-View + Multi-Scale	98.48	95.42	98.20

**Table 10:** Results showing the contribution of each component to segmentation performance.

Model Variant	Dice Score (DSC)	IoU Score
MICO_2D	0.8480	0.7361
Vese–Chan	0.8729	0.7745
LAC	0.9093	0.8337
LIF	0.9476	0.9003
Full Model (Refined Segmentation)	0.9542	0.9124

**Table 11:** Performance comparison between initial and refined semantic segmentation using DSC and IoU (INbreast dataset).

Segmentation Type	DSC	IoU
Initial Segmentation	0.904	0.825
Refined Segmentation	0.932	0.884
Difference	0.028	0.059

**Table 12:** Descriptive comparison of initial and refined segmentation performance across the dataset (median [IQR], mean difference, and bootstrap confidence intervals).

Metric	Initial (Median [IQR])	Refined (Median [IQR])	Mean Difference	Bootstrap CI (Difference)
DSC	0.789 [0.76–0.82]	0.815 [0.79–0.84]	+0.026	[0.025, 0.027]
IoU	0.679 [0.64–0.71]	0.731 [0.70–0.76]	+0.052	[0.051, 0.053]

**Table 13:** Illustrative segmentation results for a small set of INbreast images showing the effect of localized refinement. Mean ± SD and median (IQR) are reported for these representative cases.

Image ID	DSC Initial	DSC Refined	Delta DSC	IoU Initial	IoU Refined	Delta IoU
1	0.935	0.964	0.029	0.874	0.930	0.056
2	0.925	0.970	0.045	0.861	0.941	0.080
3	0.762	0.800	0.038	0.709	0.770	0.061
4	0.565	0.600	0.035	0.525	0.580	0.055
Mean ± SD	0.797 ± 0.174	0.834 ± 0.174	0.037 ± 0.007	0.742 ± 0.161	0.805 ± 0.168	0.063 ± 0.011
Median (IQR)	0.844 (0.565–0.935)	0.882 (0.600–0.970)	0.037 (0.029–0.045)	0.785 (0.525–0.874)	0.855 (0.580–0.941)	0.059 (0.055–0.080)

**Table 14:** Paired per-image *t*-statistics assessing the statistical significance of differences between initial and refined segmentation.

Metric	t-Value	*p*-Value	Holm–Bonferroni Adjusted *p*
DSC	−6.9224	$2.98\times 10^{-11}$	$2.98\times 10^{-11}$
IoU	−13.4374	$5.09\times 10^{-30}$	$1.02\times 10^{-29}$

**Table 15:** Performance comparison of multi-scale and multi-view fusion architectures for dense tissue segmentation (INbreast dataset).

Architecture	Dice (%)	IoU (%)	Sensitivity (%)
U-Net	94.40	89.70	93.00
Multi-Scale Fusion	96.21	92.90	95.70
Multi-View Fusion	96.37	93.10	96.00
Multi-View + Multi-Scale	96.53	93.51	96.20

**Table 16:** Results showing the contribution of each component to segmentation performance (INbreast dataset).

Model Variant	Dice Score (DSC)	IoU Score
MICO_2D	0.835	0.724
Vese-Chan	0.858	0.760
LAC	0.893	0.817
LIF	0.928	0.881
Full Model (Refined Segmentation)	0.932	0.884

**Table 17:** Quantitative Results of the Proposed Panoptic-Style Tumor Instance Segmentation.

Metric Category	Metric	Value
Pixel-Level	Dice Coefficient	1.0000
Pixel-Level	IoU (Jaccard Index)	1.0000
Instance-Level	Raw Tumor Instances	27
Instance-Level	Final Tumor Instances	9
Panoptic Metrics	SQ	0.8451
Panoptic Metrics	RQ	1.0000
Panoptic Metrics	PQ	0.8451

## Data Availability

The data presented in this study are available on request from the corresponding author. The dataset used in this study is publicly available. As this paper forms part of an ongoing research project, the implementation of the proposed hybrid segmentation framework and the derived annotation masks will be made publicly available on a GitHub or Kaggle repository upon completion. In the meantime, they will be provided to academic researchers upon reasonable request to support verification and extension of the results. All experimental parameters and implementation details are fully documented within the manuscript to facilitate reproducibility.
